# Sensitive detection of antigen-specific T-cells using bead-bound antigen for in vitro re-stimulation

**DOI:** 10.1016/j.mex.2019.07.004

**Published:** 2019-07-08

**Authors:** Mattias Bronge, Andreas Kaiser, Claudia Carvalho-Queiroz, Ola B. Nilsson, Sabrina Ruhrmann, Erik Holmgren, Tomas Olsson, Guro Gafvelin, Hans Grönlund

**Affiliations:** a Therapeutic Immune Design, Department of Clinical Neuroscience, Karolinska Institutet, Center for Molecular Medicine L8:02, 171 76, Stockholm, Sweden; b Neuroimmunology Unit, Department of Clinical Neuroscience, Karolinska Institutet, Center for Molecular Medicine L8:04, 171 76, Stockholm, Sweden

**Keywords:** Antigen Specific Activation of T-cells Using Bead-bound Antigens, T-cell, In-vitro assay, Antigen, Antigen processing, Microparticles

## Abstract

Reliable and sensitive detection of antigen specific cells is essential in several fields of research, whether it concerns monitoring responses to infectious agents or exploring the auto-antigen repertoire in autoimmune diseases. Identification of these cells is however difficult, especially when the cells often are rare and methods not sensitive, specific or practical enough. We propose a novel method of processing antigens before stimulation of cells which consists of covalently binding protein antigen to superparamagnetic micro-beads and using denaturing washes to remove contaminants. Peripheral blood mononuclear cells (PBMCs) from healthy donors were stimulated using both cytomegalovirus and tetanus-diphtheria antigen-beads as well as non-antigenic protein-beads as negative control in an IFNγ FluoroSpot assay in order to detect Th1 and CD8^+^ responses. The responses toward the antigen beads were both antigen specific and sensitive, with a detection threshold of 1 IFNγ producing T-cell per 18,000 PBMCs.

• Covalently binding antigen to paramagnetic beads allows for harsh denaturing washes without loss of antigen.
• Microbeads are phagocytosed by antigen presenting cells, resulting in efficient uptake, processing and presentation of the antigens.
• The method allows the usage of relatively impure starting antigen material and whole PBMC samples without high background levels in follow up cellular assays.

## Graphical abstract

**Figure:**
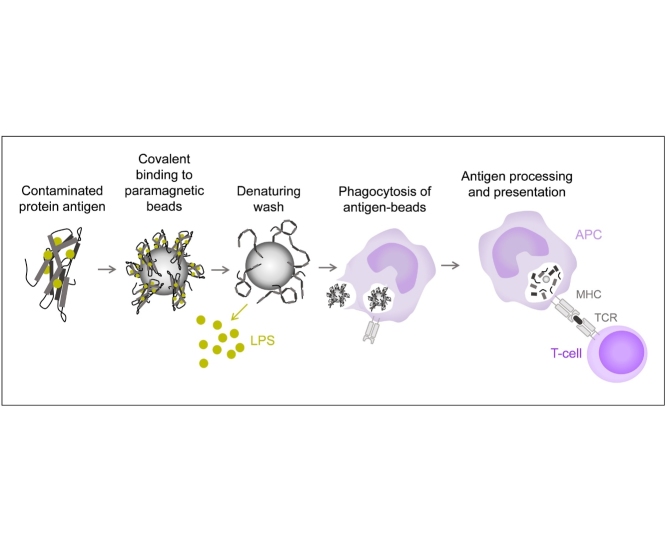

**Specifications Table**

**Table:** 

Subject Area:	Immunology and Microbiology
More specific subject area:	Detection of antigen-specific T-cells
Method name:	Antigen Specific Activation of T-cells Using Bead-bound Antigens
Name and reference of original method:	[1] M. Bronge, S. Ruhrmann, C. Carvalho-Queiroz, O. B. Nilsson, A. Kaiser, E. Holmgren et al. Myelin oligodendrocyte glycoprotein revisited—sensitive detection of MOG-specific T-cells in multiple sclerosis. J Autoimmun, 2019.
Resource availability:	Paramagnetic Beads (Dynabeads MyOne, carboxylic acid, ThermoFisher Scientific, USA) FluoroSpot (Pre-coated FluoroSpot kit, Mabtech, Sweden) and a FluoroSpot reader

## Method details

### Methodology background

Identification of antigen-specific T-cells is an important aspect of several different areas of immunological research, from infections to autoimmune diseases and cancer immunology. There are several different methods available for detecting antigen-specific T-cell responses, such as cell-proliferation assays, ELISpot/FluoroSpot or flow-cytometry based assays. There are advantages and disadvantages with each method, but problems often arise due to assays having a high background noise owing to an abundance of non-specific responses while the cells specific for the antigen in question are rare [2]. The challenge can increase during the production of the antigens. For example *E. coli* expression of recombinant proteins include high amounts of bacterial contaminants like lipopolysaccharide (LPS), which can be difficult to remove and will interfere in follow-up cellular assays [3]. This is increasingly problematic when the antigen of interest is unstable or insoluble. Using shorter synthetic peptides forego this problem but at the cost of lower sensitivity, and are only practical to use when testing for a limited amount of previously known or suspected T-cell epitopes. Particles in the micro- and nanometer size range have previously been studied as possible adjuvants in vaccination due to their ability to efficiently induce antigen specific responses in vivo [4,5]. Using particles, particularly in the 1 μm range, as a vehicle for antigen results in efficient phagocytosis, processing and presentation by antigen-presenting cells [[6], [7], [8]]. These properties, in combination with the possibility of covalently coupling protein to paramagnetic beads which in turn enables harsh denaturing washes to remove bacterial contaminants without loss of the protein, allowed the development of an antigen-processing method using paramagnetic beads for in vitro activation and detection of antigen-specific T-cells.

### Sample collection

Venous blood samples were drawn from healthy donors (HDs) (n = 28) using EDTA tubes (BD diagnostics) and peripheral blood mononuclear cells (PBMCs) were isolated via density gradient centrifugation (Ficoll-Plaque plus, GE healthcare) in the SepMate system (Stemcell Technologies) as per manufacturers protocol. Remaining red blood cells were lysed by ACK-lysing buffer (Sigma-Aldrich) for 5 min before the cells were washed and resuspended in freezing medium (45% RPMI 1640 (Sigma-Aldrich), 45% heat inactivated FCS and 10% DMSO) at RT at a concentration of 10–15 × 10^6^ cells/ml. The PBMCs were then frozen at −1 °C/min using a CoolCell (Biocision, USA) and subsequently stored at −150 °C. All donors gave their written informed consent before participating. The study was approved by the regional ethics board, Regionala Etikprövningsnämnded in Stockholm and was conducted in accordance with the Declaration of Helsinki principles.

### Design of antigens

**Fig. 1:**
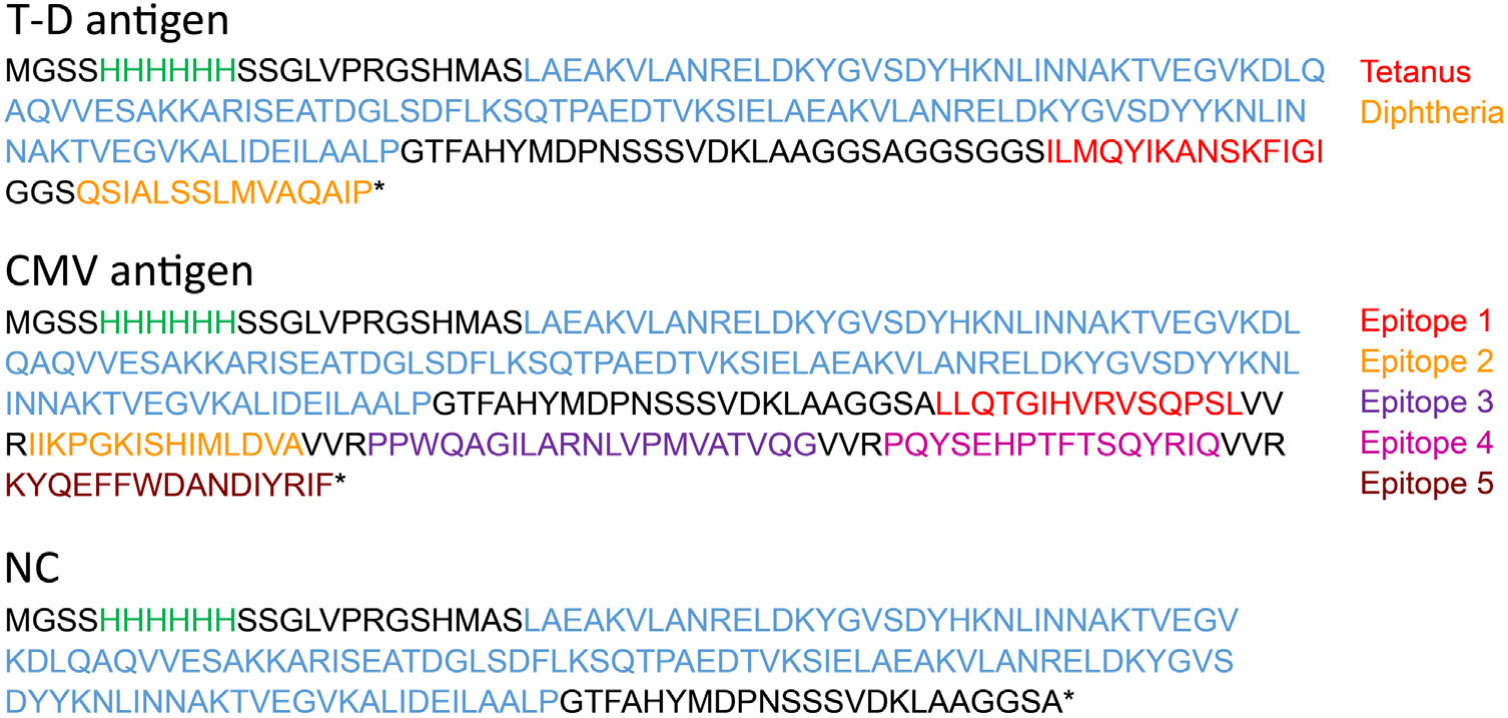
Design of antigens. Amino acid sequences of the Tetanus-Diphtheria-antigen and Cytomegalovirus-antigen, containing a 6xHIS-tag for purification and ABD for quality control purposes. Linker sequences in black, histidine tag in green and albumin binding domain in blue. T-D: Tetanus-Diphtheria. CMV: Cytomegalovirus. NC: Negative control.

A combination of epitopes from tetanus toxin (T) and diphtheria toxin (D), as previously described [9], with an interspacing GGS linker (T-D antigen) and a combination of five epitopes from the Cytomegalovirus protein PP65 [10] (CMV-antigen) were designed. The genes were subcloned using a one-step digestion-ligation reaction with BsaI and T4 DNA ligase into a modified pET28a expression vector (Merck-Millipore) containing an albumin binding domain (ABD) [11]. The resulting protein sequences contained an N-terminal 6x histidine purification tag, ABD and T-D or CMV-epitopes (Fig. 1). As a negative control (NC), the histidine tag and ABD with linkers was produced. The finished vectors were transformed into *E. coli* BL21-AI (ThermoFisher Scientific).

### Expression and purification of the antigens

Transformed *E. coli* were grown in 500 ml medium supplemented with kanamycin, glycerol, Mg2S04, arabinose, lactose and glucose for auto-induction at 25 °C overnight. Cells were centrifu ged at 9000 *g* and lysed using a 6 M Guanidinium-HCl, 10 mM Tris-HCl, 50 mM NaHPO4, 100 mM NaCl and 20 mM β-mercaptoethanol, pH 8.0 buffer followed by freezing at −80 °C. Supernatants were collected after centrifugation at 23,500 *g*. The antigens were purified using His Mag Sepharose Ni Beads (GE Healthcare) and eluted with a 6 M Urea, 0.05% Tween 20, 10 mM β-mercaptoethanol, 10 mM MES, pH 3.0 buffer. The antigens were adjusted to pH 5.0 by addition of 0.82 M MES buffer pH 6.0.

### Antigen bead coupling

The protein antigens (CMV, T-D and NC) were covalently linked to 1 μm paramagnetic polystyrene beads (Dynabeads MyOne Carboxylic acid, Thermofisher) using amine coupling. A magnet (Magrack 6, GE) was used to facilitate recovery of the beads between each following steps. To keep the beads in suspension all incubation steps were performed in 1.5 ml tubes on a shaker block at ≥900 RPM. In order to minimize bead losses when handling the beads, low retention tubes and pipette tips were used throughout the whole procedure. The beads were first washed twice in 25 mM MES buffer, pH 6, followed by incubation for 30 min at room temperature with 0.1 M *N*-hydroxysuccinimide (NHS) (Sigma-Aldrich) and 0.4 M 1-ethyl-3-(3-dimethylaminopropyl)carbodiimide (EDC) (Fisher Scientific) in MES-buffer. After washing twice in MES-buffer, the antigen was added at a concentration of 1 mg/ml in MES-buffer, 67 μg protein/mg (n = 10^9^) beads, and incubated for 30 min at room temperature. After antigen coupling, still reactive carboxylic groups were blocked by incubating with 50 mM Tris pH 7.4 for 15 min.

### Coupling quality control

**Fig. 2:**
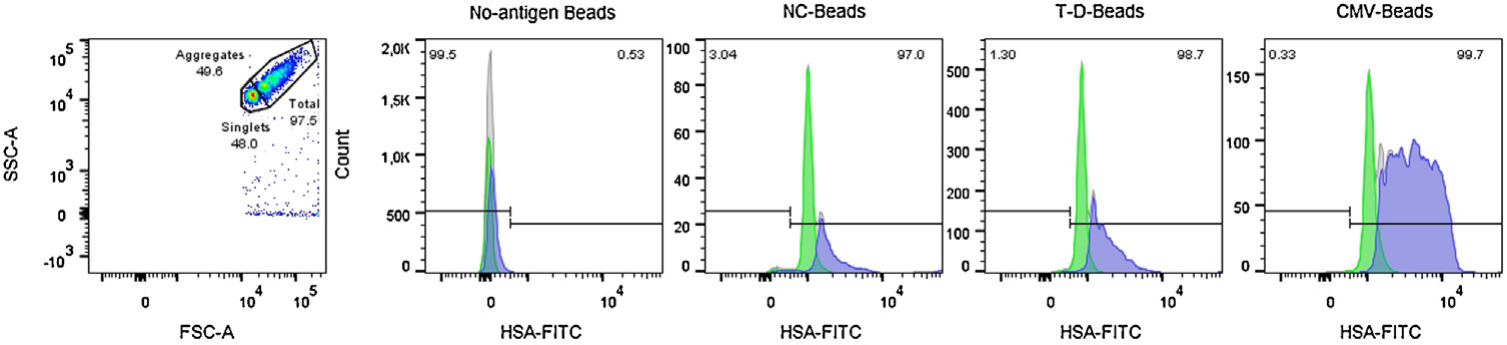
Quality control of the antigen-bead coupling using flow cytometry. The albumin-binding tag used as a negative control was also included in the antigen-constructs, and used to stain the beads with fluorophore-conjugated human serum albumin. The beads were first sorted into single-, aggregate- and total-gates based on forward- and side-scatter (leftmost plot consists of representative example from the no-antigen beads). Based on the no-antigen beads, a cutoff for positivity was defined (second diagram from the left) and then applied for the antigen-beads. As shown, the single gates all stained homogenously and positive for the antigen. Total beads gate are shown in grey, single beads in green and aggregated beads in blue.

In order to be able to ensure a successful coupling, we opted to include an albumin binding tag, both as the negative control (NC-beads) and in the CMV- and T-D- antigen constructs. This allowed for staining the bead-bound antigens using a fluorophore-conjugated human serum albumin (HSA). In this method, ˜10^6^ beads were incubated with 5 μl FITC-HSA (Abcam) for 30 min at RT and subsequently washed twice in PBS before acquisition in a FACSVerse (BD biosciences, USA). Using non-antigen beads as a negative control, the number of beads containing a significant amount of the antigen was gated using flow cytometry software (FlowJo 10, FlowJo LCC, USA) (Fig. 2). While this method did not give the absolute amount of antigen coupled, the different antigens could be compared to ensure acceptable homogeneity.

### Endotoxin removal

**Fig. 3:**
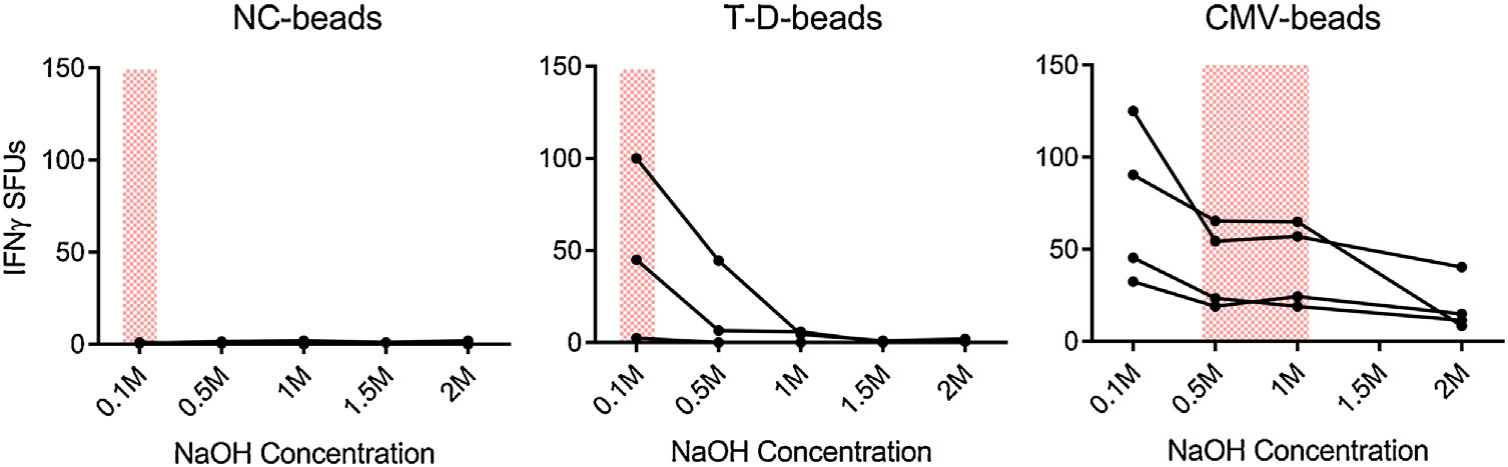
Test of different NaOH-concentrations required for endotoxin removal. Antigen-beads were washed with different concentrations of NaOH and tested for induction of IFNγ production of healthy donor (n = 3–4) PBMCs by IFNγ FluoroSpot. Red-areas indicate which wash-conditions were deemed optimal. NC: Negative control. T-D: Tetanus-Diphtheria. CMV: Cytomegalovirus SFUs: Spot forming units.

As an integral part of the antigen preparation, the beads were washed with an NaOH solution to remove endotoxin. As different proteins can be expected to contain different amounts of endotoxin, and trace amounts of LPS can facilitate antigen-specific responses [3], the optimal NaOH concentration was determined individually for each antigen bead. The beads were washed with 0.1–2 M NaOH and used as stimuli in an IFNγ FluoroSpot assay (Fig. 3). The optimal washing condition was determined as the NaOH concentration which resulted in the highest signal to noise ratio. In this case, 0.1 M for the NC- and T-D-beads and 0.75 M for the CMV-beads were chosen. For the washing procedure, the NaOH-solution was added to the beads, the beads were vortexed gently and the supernatant was discarded. The procedure was repeated four times followed by five washes in sterile PBS. The antigen-beads were then stored in −80 °C.

### Endotoxin test

The remaining endotoxin on the beads was tested using a limulus-amebocyte-lysate-assay (Endpoint endochrome kit, R160K, Charles River) according to the manufacturer’s protocol. The beads were diluted to 0.4–1 × 10^9^ beads/ml and incubated at 95 °C for 5 min before testing. The FBS used in the cell culture media in the FluoroSpot assay was similarly tested. All tests were done in duplicates in a 96-well plate and LPS-concentration was determined by OD at 405 nm. The rationale for briefly incubating the beads at 95 °C prior to testing was to release as much endotoxin as possible from inside folded proteins. Testing the beads together with the supernatant increased the sensitivity of the assay, while the beads themselves did not interfere with the OD-reading.

### FluoroSpot assay

To assess the T-cell responses towards the antigen-beads an IFNγ FluoroSpot assay (FSP-011803, Mabtech, Sweden) was used. PBMCs were thawed in a water bath at 37 °C before being washed twice in cRPMI (RPMI 1640 (Sigma-Aldrich) with 10% FBS (lot F7524, Sigma-Aldrich), 2 mM l-Glutamine (G7513, Sigma-Aldrich), 100U/ml penicillin and 100 μg/ml streptomycin (P4333, Sigma-Aldrich)). PBMCs were plated in a 96 well FluoroSpot plate at 2.5 × 10^5^ PBMCs/well and antigen beads were added in duplicates to a concentration of 10 beads per PBMC. The plates were incubated at 37 °C, 5% CO_2_ for 44 h before development according to manufacturer’s protocol. The plates were read and spot forming units (SFUs) counted using an iSPOT Spectrum (AID, Germany). As certain antigen beads can be sticky, and small amounts can be lost due to contact with plastic, we found that keeping them in high concentration (10 mg/ml, or 10^10^/ml) minimizes the loss. In order to get a manageable concentration for pipetting during the FluoroSpot assay, the beads are preferentially diluted in cRPMI before adding to the wells, as cRPMI reduces the stickiness. Our titration trials indicate that ˜10 beads per PBMC give the best separation between antigen responsive individuals and background in the FluoroSpot assay, however differences have been seen in the range of 2.5–20 beads per PBMC.

### Statistical analysis

Statistical analyses were performed using GraphPad Prism 7 (GraphPad Software, USA). A Mann-Whitney *U* test was used to compare the responses to the antigen-beads and negative control beads. A Spearman correlation was used to correlate the individual CMV and T-D responses. Before this correlation, each individual’s background response was subtracted from the antigen response (ΔSFU). P < 0.05 was set as significance threshold for all analyses.

## Method validation

**Fig. 4:**
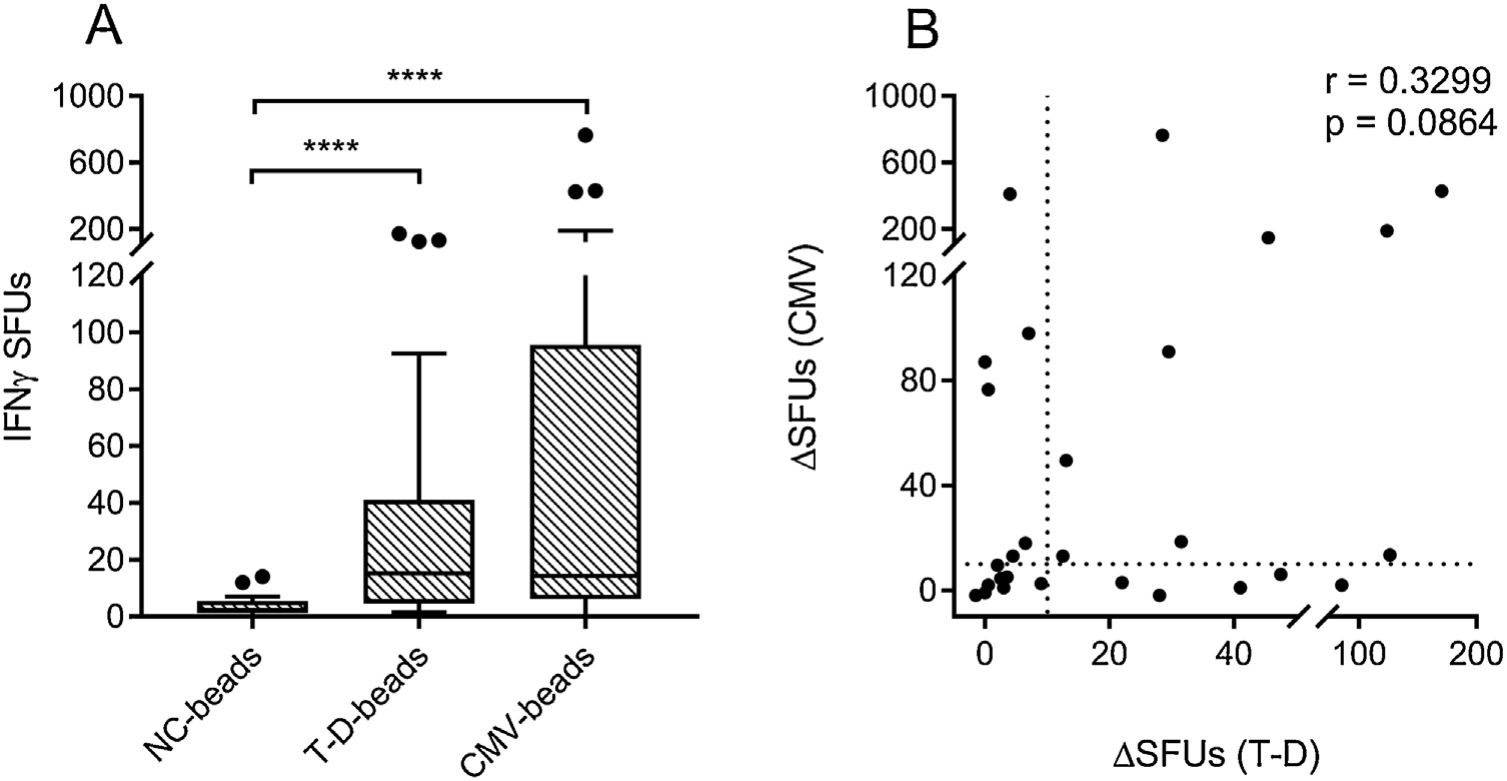
PBMC responses towards antigen beads. T-D and CMV antigen beads were used to stimulate PBMCs from healthy donors (n = 28) in an IFNγ FluoroSpot assay. A. IFNγ SFUs in response to negative control-, T-D- and CMV-beads. Box represent median and IQR, bars 1.5x IQR (tukey). P-values calculated with Mann-Whitney *U* test. ****p < 0.0001. B. Correlation between individual T-D and CMV responses. P-values calculated with Spearman correlation. Dotted lines indicate threshold for positivity, calculated as +3SD of the NC-responses. NC: Negative Control. T-D: Tetanus-Diphtheria. CMV: Cytomegalovirus. SFUs: Spot forming units. ΔSFUs: Spot forming units above negative control.

After the NaOH wash, the levels of endotoxin contamination of the beads were very low (Table 1). All antigen beads contained significantly less endotoxin than the FBS used in the cell-medium when adjusted for the amount used. To confirm that the beads activated T-cells with low background, we performed an IFNγ FluoroSpot assay using PBMCs from HDs stimulated with CMV-antigen-beads, T-D-antigen-beads or negative-control-beads (Fig. 4A). A negligible activation was seen in response to the negative control beads with a mean ± SD number of spot forming units of 3.5 ± 3.4 per 2.5 × 10^5^ PBMCs, indicating a very low background noise level. Both T-D-beads and CMV-beads gave rise to a significantly higher response than the background (33.8 ± 44.6 and 90.9 ± 174.3 SFUs respectively, p < 0.0001), indicating a targeted response towards these antigens. To see whether there was a discrepancy in individuals’ responses to T-D and CMV-beads, indicating antigen specificity, the individual HD responses towards CMV and T-D were correlated (Fig. 4B), showing that there was not a significant correlation between the two (p > 0.05). As different individuals have different background responses, each individual’s background SFUs was subtracted from the CMV or T-D induced SFUs (resulting in ΔSFUs) before performing the correlation analysis. Additionally, using a cutoff for positivity as +3SD of the negative control (≥10.5 ΔSFUs), the responses could be separated into T-D^+^CMV^+^ (n = 9, 32.1%), T-D^+^CMV^−^ (n = 5, 17.9%), T-D^−^CMV^+^ (n = 6, 21.4%) and T-D^−^CMV^−^ (n = 8, 28.6%) populations. Using a mean+3SD above the NC-response as the limit for detection, the assay was able to detect antigen specific cell responses as rare as 1 IFNγ producing T-cell per 18,000 PBMCs. The mean SFUs of antigen-responsive and antigen-non-responsive individuals were 61.2 vs 6.4 for the T-D beads and 165.0 vs 5.6 for the CMV-beads, giving a signal to noise ratio of 9.6 and 29.5 respectively. The validation was based on FluoroSpot as the follow-up assay. While the FluoroSpot/ELISpot assay is very sensitive as it detects responses in single cells and might thus be optimal when looking for rare antigen-specific responses, the use of antigen-beads instead of soluble antigen as stimuli can likely be used in other cellular methods as well. Additionally, this validation used IFNγ production as a measurement of response, meaning it mainly detected Th1 and CD8^+^ cells. In order to detect other types of antigen-specific responses which could be more relevant to the study in question, one must combine this antigen-processing method with other readouts, e.g. IL-10, TNFα or IL-17 production or cell-proliferation.

**Table 1:** Endotoxin contamination of the antigen-beads after NaOH-washing.

	LPS per antigen-bead (EU/10^9^ beads)	LPS from source in the FluoroSpot-assay (EU/ml)
NC-Beads	0.088	0.0011
T-D-Beads	0.59	0.0074
CMV-Beads	0.069	0.00086
Fetal bovine serum	–	>0.06 (LAL-test)
		<1.0 (Supplier guarantee)

*LPS*: Lipopolysaccharide, *NC*: Negative Control, *T-D*: Tetanus-Diphtheria, *CMV*: Cytomegalovirus, *LAL*: Limulus-amebocyte-lysate.

The study has been partly funded by grants from CBD solutions, Neuroförbundet, Swedish Research Council (2017-00777), Swedish Brain Foundation, Margareta af Ugglas Foundation and Stratneuro. None of the funding sources have had a role in the study design, collection, analysis or interpretation of the data.

## Declaration of Competing Interest

We wish to draw the attention of the editor to the following facts which may be considered as potential conflicts of interest. There are pending patents regarding parts of the methods presented in this manuscript, more precisely the usage of antigen-beads for detection of antigen-specific T-cells. Mattias Bronge and Hans Grönlund are the inventors of said patents which are held by the private company TCER AB. Hans Grönlund is a co-founder of TCER AB and Claudia Carvalho-Queiroz, Ola Nilsson and Andreas Kaiser hold positions at TCER AB.

The study has been partly funded by grants from CBD solutions, Neuroförbundet, Swedish Research Council (2017-00777), Swedish Brain Foundation, Margareta af Ugglas Foundation and Stratneuro. None of the funding sources have had a role in the study design, collection, analysis or interpretation of the data.
