# Supplementary material for: Sensitive detection of antigen-specific T-cells using bead-bound antigen for in vitro re-stimulation
Source: MethodsX. 2019 Jul 8;6:1635–41. doi: 10.1016/j.mex.2019.07.004 (PMC6651840; doi:10.1016/j.mex.2019.07.004)
Supplement: Supplementary file 1 [file mmc1.docx]

**Additional information:**

Sample collection

PBMC collection using EDTA-blood collection tubes and a follow-up density gradient centrifugation with Ficoll-Plaque is superior to using already-prepared cell preparation blood collection tubes (i.e. Vacutainer CPT tubes) for follow-up responses using antigen beads. The reason for this is however unknown.

Endotoxin wash

For some antigens (not shown in this study) 2M NaOH has been insufficient in reducing the background. In these cases, an additional wash procedure with PBS 1% Triton X-100 has been performed. Contrary, using the harshest wash for all antigens carries the risk of removing too much contaminants, some of which are probably needed as co-stimulators in follow-up assays for facilitating the antigen-specific responses. The T-D-beads used in this study are an example of this. Thus, the optimal condition for reaching the largest signal to noise ratio has to be titrated for each individual assay and antigen.

Coupling QC

Indirect protein quantification by bicinchoninic acid (BCA) assay or light absorbance of the supernatant after bead-coupling and back-calculation of the amount of coupled protein was not feasible due to interference with the coupling chemicals (EDC and NHS) with these methods.
